# Deubiquitinating enzymes: potential regulators of the tumor microenvironment and implications for immune evasion

**DOI:** 10.1186/s12964-024-01633-7

**Published:** 2024-05-07

**Authors:** Sheng-Kai Hsu, Chon-Kit Chou, I-Ling Lin, Wen-Tsan Chang, I-Ying Kuo, Chien-Chih Chiu

**Affiliations:** 1 Department of Biotechnology, Kaohsiung Medical University, Kaohsiung, 807 Taiwan; 2 State Key Laboratory of Quality Research in Chinese Medicine, Institute of Chinese Medical Science, University of Macau, Macau SAR, 999078 P.R. China; 3 Department of Medical Laboratory Science and Biotechnology, Kaohsiung Medical University, Kaohsiung, 807 Taiwan; 4 Division of General and Digestive Surgery, Department of Surgery, Kaohsiung Medical University Hospital, Kaohsiung, 807 Taiwan; 5 Department of Surgery, School of Medicine, College of Medicine, Kaohsiung Medical University, Kaohsiung, 807 Taiwan; 6 Center for Cancer Research, Kaohsiung Medical University Hospital, Kaohsiung Medical University, Kaohsiung, 807 Taiwan; 7 Department of Biological Sciences, National Sun Yat-Sen University, Kaohsiung, 804 Taiwan; 8 Department of Medical Research, Kaohsiung Medical University Hospital, Kaohsiung, 807 Taiwan

**Keywords:** Deubiquitinating enzymes (DUBs), Tumor microenvironment (TME), Immune evasion, Posttranslational modification (PTM), Tumor-associated macrophage (TAM), Regulatory T cell (Treg), Cytotoxic T cell (CTL)

## Abstract

Ubiquitination and deubiquitination are important forms of posttranslational modification that govern protein homeostasis. Deubiquitinating enzymes (DUBs), a protein superfamily consisting of more than 100 members, deconjugate ubiquitin chains from client proteins to regulate cellular homeostasis. However, the dysregulation of DUBs is reportedly associated with several diseases, including cancer. The tumor microenvironment (TME) is a highly complex entity comprising diverse noncancerous cells (e.g., immune cells and stromal cells) and the extracellular matrix (ECM). Since TME heterogeneity is closely related to tumorigenesis and immune evasion, targeting TME components has recently been considered an attractive therapeutic strategy for restoring antitumor immunity. Emerging studies have revealed the involvement of DUBs in immune modulation within the TME, including the regulation of immune checkpoints and immunocyte infiltration and function, which renders DUBs promising for potent cancer immunotherapy. Nevertheless, the roles of DUBs in the crosstalk between tumors and their surrounding components have not been comprehensively reviewed. In this review, we discuss the involvement of DUBs in the dynamic interplay between tumors, immune cells, and stromal cells and illustrate how dysregulated DUBs facilitate immune evasion and promote tumor progression. We also summarize potential small molecules that target DUBs to alleviate immunosuppression and suppress tumorigenesis. Finally, we discuss the prospects and challenges regarding the targeting of DUBs in cancer immunotherapeutics and several urgent problems that warrant further investigation.

## Introduction

Ubiquitination is a tightly controlled posttranslational modification in which ubiquitin is attached to target proteins, and it is reportedly involved in several biological processes, such as proliferation, differentiation, apoptosis, and the immune response [1]. Ubiquitination is mediated by a series of enzymes, including ubiquitin-activating enzymes (E1s), ubiquitin-conjugating enzymes (E2s), and ubiquitin ligases (E3s) [2]. The opposing process, deubiquitination, is reversible and involves the deconjugation of ubiquitin moieties from protein substrates via multiple deubiquitinating enzymes (DUBs) [3]. Human DUBs belong to a protein superfamily that consists of seven subfamilies with more than 100 members divided into two classes based on sequence similarity and domain conservation: cysteine proteases and metalloproteases. There are six cysteine protease subfamilies, including Machado–Joseph domain-containing proteases (MJDs), motif interacting with Ub-containing novel DUBs (MINDYs), ovarian tumor proteases (OTUs), ubiquitin carboxy-terminal hydrolases (UCHs), ubiquitin-specific proteases (USPs), and Zn-finger and UFSP domain proteins (ZUFSPs). JAMM/MPN domain-associated metallopeptidases (JAMMs) are the only metalloproteases of this family [4, 5]. Physiologically, the balance between ubiquitination and deubiquitination is important for maintaining cellular homeostasis, as it strictly regulates protein turnover. However, dysregulation of DUBs has been reported in multiple cancers and is suggested to promote tumorigenesis-related processes and phenotypes such as angiogenesis, metastasis and therapeutic resistance [6–9].

The tumor microenvironment (TME) is a highly dynamic and complex entity comprising not only tumor cells but also noncancerous cells (e.g., diverse immune cells and cancer-associated fibroblasts (CAFs)) and acellular components (e.g., the extracellular matrix (ECM)). Once considered bystanders, these tumor-surrounding components are largely involved in tumor progression and have recently been identified as attractive therapeutic targets [10]. The emergence of immune checkpoint blockade (ICB) therapy has remarkably revolutionized cancer treatment [11]. Unfortunately, the response rate to ICB is unsatisfactory, particularly in solid tumors, largely because of the immunosuppressive nature of the TME. Natural killer (NK) cells and CD8^+^ T lymphocytes are the main effector cells involved in the antitumor immune response [12]; however, their functions or proximity to the tumor compartment are often limited by infiltrating immunosuppressive cells, including tumor-associated macrophages (TAMs), myeloid-derived suppressor cells (MDSCs) and regulatory T cells (Tregs) [13, 14]. Their interplay not only facilitates cancer immune evasion but also decreases the efficacy of ICB. Although the role of CAFs in tumorigenesis is controversial due to their high heterogeneity [15], a large body of evidence still indicates that they contribute to ICB-related failure by sequestering CD8^+^ T cells [16–18]. Hence, exploring therapeutic strategies to modulate the interplay of TME components could improve the response to immunotherapy in the context of cancer treatment.

Recently, emerging evidence has demonstrated the involvement of DUBs in immunosuppression: they regulate immune checkpoints (e.g., PD-L1 and PD-1) expression or inhibit effector immune cell recruitment and function [19–22]. For example, macrophage-intrinsic ubiquitin-specific protease 1 (USP1) inhibits the recruitment of CD8^+^ T cells and simultaneously promotes colorectal cancer (CRC) stemness and liver metastasis [19]. Therefore, targeting dysregulated DUBs seems promising for restoring immunosurveillance and improving the efficacy of ICB therapy in suppressing tumor progression. In this review, we elucidate the roles of DUBs in the dynamic interplay between tumors, immune cells, and stromal cells. We also summarize DUBs as potential therapeutic targets for restoring antitumor immunity and restraining tumorigenesis. Finally, the advantages and limitations of targeting DUBs for cancer immunotherapeutics are elaborated.

## The interplay between DUBs and the tumor microenvironment

Emerging findings have revealed that tumor- or TME component-intrinsic DUBs orchestrate the dynamic interplay within the TME, which further promotes tumor progression and facilitates immunosuppression [20, 23–26]. Among these cells, NK and CD8^+^ T cells are the main immune effector cells that exert antineoplastic effects [12]. In contrast, TAMs, MDSCs and Tregs are well-recognized immunosuppressive cells [27]. Additionally, CAFs reportedly drive tumorigenesis [28]. Hence, in the following sections, we discuss the involvement of DUBs in the crosstalk between tumors and these TME components and summarize potential small molecules that target DUBs to suppress tumor progression or synergize with ICB by modulating the TME.

## DUBs-mediated suppression of NK function and infiltration

NK cells, key effector cells of innate immunity, execute serial killing events on target cells through granzyme B (GrB), perforin and death receptor-mediated signaling [29]. Additionally, NK cells secrete cytokines to recruit or induce other immune cells to bridge innate and adaptive immunity [30, 31]. Death-associated protein kinase 1 (DAPK1), a tumor-suppressive serine/threonine kinase, is involved in apoptosis and autophagy [32], and a recent study suggested that its dysregulation causes NK cell exhaustion in a DUB-dependent manner [33]. Mechanistically, the downregulation of DAPK1 facilitates IKKβ-mediated COP9 signalosome 5 (CSN5) phosphorylation and further deubiquitinates PD-L1, which compromises NK cell-mediated killing effects [33]. Furthermore, USP10 desensitizes pancreatic ductal adenocarcinoma (PDAC) cells to NK cell-mediated cytotoxicity by deubiquitinating yes-associated protein 1 (YAP1, a core component of Hippo signaling) to transcriptionally upregulate both PD-L1 and galectin-9 (Gal-9) [23]. Gal-9 is an immune checkpoint that decreases NK cell activity and even promotes immune tolerance [34]. Increased infiltration of NK cells within the TME is a favorable prognostic factor in several solid tumors [35]; however, USP22 has been reported to suppress NK cell infiltration by altering the transcriptome of pancreatic cancer (PC) cells [36]. Overall, these findings indicate that the inhibition of DUBs may facilitate NK-cell-mediated tumor clearance by enhancing NK-cell activity and infiltration.

### The role of DUBs in TAM-mediated immunosuppression

Macrophages are important for inflammation and are responsible for tissue repair and homeostasis. Macrophage polarization refers to the process in which macrophages are driven to a specific phenotype by proinflammatory stimuli within the TME. Macrophages are polarized into two phenotypes, M1 and M2. M1 macrophages have proinflammatory, tissue-damaging and antitumor effects; conversely, M2 macrophages have anti-inflammatory, tissue repair-related and protumor effects [37, 38]. TAMs, which are enriched within the TME, are estimated to account for 30 ~ 50% of infiltrating cells [39] and reportedly lead to tumor progression, including therapeutic resistance, metastasis, and immunosuppression [40]. To this end, several therapeutic strategies have been proposed to suppress the tumor-promoting effects mediated by TAMs: (1) depleting TAMs from tumor tissues, (2) reprogramming TAMs to the antitumor phenotype, and (3) blocking the recruitment of M2 macrophages. Depletion of TAMs could inhibit immunosuppression but might also significantly decrease the number of M1 macrophages and even tissue-resident macrophages (e.g., microglia and Kupffer cells) [41]. Hence, among these strategies, modulating TAM reprogramming and infiltration to reverse immunosuppression has recently attracted much attention.

**Fig. 1:**
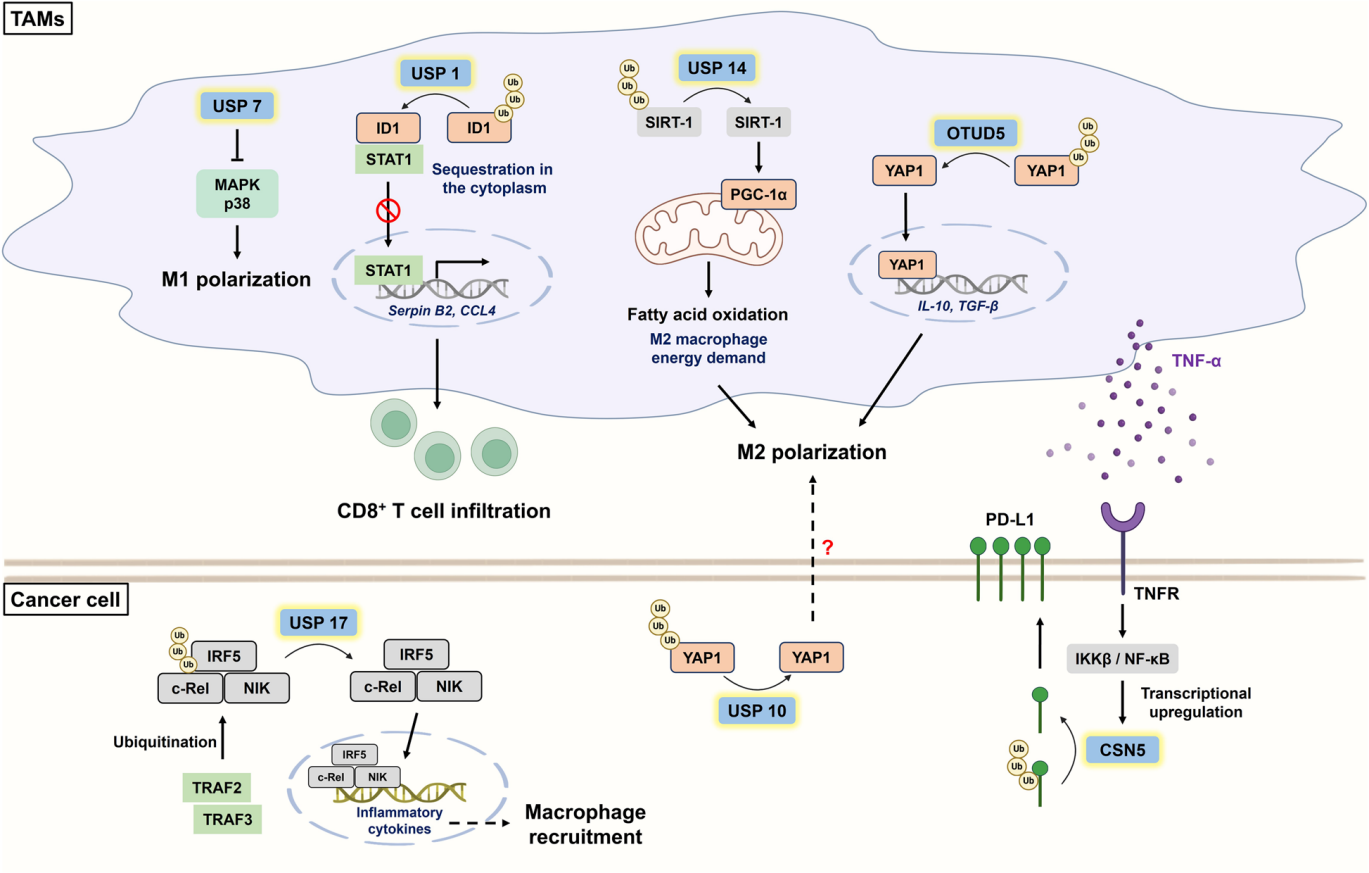
The involvement of DUBs in the crosstalk between tumors and tumor-associated macrophages (TAMs). TAMs are enriched within the TME and are largely involved in tumorigenesis and immunosuppression. Within TAMs, USP7 blocks p38 MAPK-induced M1 polarization. However, USP14 and OTUD5 facilitate M2 polarization by promoting SIRT-1/PGC-1α-mediated FAO and deubiquitinating YAP1, respectively. USP1 enhances the stability of ID1, subsequently restraining STAT1 in the cytoplasm and downregulating CD8^+^ T-cell-recruiting factors (e.g., Serpin B2 and CCL4). USP17 counteracts TRAF2/3-mediated ubiquitination of IRF5, c-Rel, and NIK and thus increase macrophage recruitment. Additionally, TAM-derived TNF-α upregulates CSN5 via IKKβ/NF-κB to promote PD-L1 stability and cause CD8^+^ T-cell exhaustion. Tumor-intrinsic USP10 might drive M2 polarization via the YAP1/IL-6 axis. CCL4: C-C motif chemokine ligand 4; CSN5: COP9 signalosome 5; ID1: inhibitor of differentiation 1; IRF5: interferon regulatory factor 5; NIK: NF-κB-inducing kinase; OTUD5: OTU deubiquitinase 5; PGC-1α: peroxisome proliferator-activated receptor-γ coactivator 1α; SIRT-1: sirtuin 1; STAT1: signal transducer and activator of transcription 1; TNFR: tumor necrosis factor receptor; TRAF2: TNF receptor associated factor 2; TRAF3: TNF receptor associated factor 3; Ub: ubiquitin; YAP1: yes-associated protein 1

Accumulating evidence has revealed the involvement of DUBs in regulating macrophage polarization. OTU deubiquitinase 5 (OTUD5) and USP10 can stabilize YAP1 to induce M2 polarization, subsequently increasing the metastatic capacity of triple-negative breast cancer (TNBC) and PDAC cells, respectively [23, 25]. However, the mechanisms of M2 polarization triggered by these two DUBs are completely different. On the one hand, macrophage-intrinsic OTUD5 deubiquitinates YAP1 to transcriptionally upregulate M2-related cytokines, such as IL-10 and TGF-β, and YAP1^+^ TAMs further promote TNBC metastasis via the CCL2/CCR2 axis [25]. Indeed, CCL2 not only drives tumor progression but also serves as an M2 recruiter and stimulator [42]. On the other hand, coincubation of differentiated THP-1 cells with USP10-knockdown PDAC cells reduced the number of M2 macrophages, but the underlying mechanism was not identified [23]. This could be explained by IL-6-mediated M2 polarization via dysregulation of YAP [43, 44] **(**Fig. 1**)**. Interestingly, a recent finding revealed that USP14 induces M2 polarization through the reprogramming of macrophage metabolism [45]. Notably, M1 macrophages depend on glycolysis, whereas M2 macrophages mainly rely on fatty acid oxidation (FAO) for energy demand [46, 47]. Macrophage-intrinsic USP14 deubiquitinates and stabilizes sirtuin-1 (SIRT-1) to induce peroxisome proliferator-activated receptor-γ coactivator 1α (PGC-1α) signaling and promote FAO, which results in immunosuppressive phenotypes **(**Fig. 1**)**. Conversely, the administration of IU1 (a USP14 inhibitor) effectively suppresses FAO-induced M2 polarization and tumor growth in gastric cancer (GC) [45] **(**Table 1**)**. In addition to regulating TAM reprogramming, DUBs regulate the infiltration of M2 macrophages. Upregulation of lung cancer-intrinsic USP17 (also known as DUB3) induced by TAM-secreted inflammatory stimuli protects interferon regulatory factor 5 (IRF5), c-Rel, and NF-κB-inducing kinase (NIK) from TRAF2/3-mediated ubiquitin‒proteasome degradation, which not only promotes M2 macrophage recruitment to the TME but also forms a positive feedback loop to continuously induce USP17 expression [48] **(**Fig. 1**)**. WP-1130 was reported to decrease the enzymatic activity of USP17 and inhibit breast cancer (BC) metastasis by promoting Snail degradation [49], suggesting its potential to relieve immunosuppression in lung cancer.

Previous research revealed that TAMs facilitate immune escape by suppressing CD8^+^ T-cell infiltration and function, which contributes to cancer progression-related phenotypes and processes, including chemoresistance and metastasis [14, 50], which illustrates the close interplay between TAMs and CD8^+^ T cells. In line with these findings, cumulative studies have suggested that TAMs can suppress the infiltration and decrease the activity of CD8^+^ T cells in a DUB-dependent manner. Within macrophages, USP1 deubiquitinates inhibitor of differentiation 1 (ID1) to sequester signal transducer and activator of transcription 1 (STAT1) in the cytoplasm, resulting in transcriptional downregulation of CD8^+^ T-cell-recruiting factors (e.g., CCL4 and SerpinB2) **(**Fig. 1**)**. Notably, ID1 is a substrate of USP1 [51], and blockade of USP1 by ML323 (a selective inhibitor of the USP1-UAF1 complex) not only promoted CD8^+^ T-cell infiltration and function but also markedly inhibited the liver metastasis of CRC in an orthotopic liver metastatic syngeneic mouse model [19] **(**Table 1**)**. USP7 is particularly highly expressed in M2 macrophages, and USP7 inhibition reprogrammed macrophages toward the M1-like phenotype (indicated by a significant increase in the M1/M2 macrophage ratio) by initiating the p38 MAPK signaling pathway **(**Fig. 1**)**. In vivo, blockade of USP7 by P5091 facilitated the recruitment of IFN-γ^+^ CD8^+^ T cells, accompanied by PD-L1 downregulation, which synergized with ICB to retard Lewis lung cancer growth [52] **(**Table 1**)**. Remarkably, P5091 specifically targets USP7 by blocking its active site instead of affecting its expression [53]. As mentioned above, PD-L1 can be deubiquitinated by CSN5 to enhance its stability. TAM-mediated secretion of TNF-α activates IKKβ/NF-κB/CSN5 signaling to increase PD-L1 levels, while curcumin suppresses this effect by counteracting CSN5-related kinase activity [54, 55] **(**Fig. 1**)**. In addition, curcumin synergizes with an anti-CTLA-4 mAb to potently boost CD8^+^ T-cell-mediated cytotoxicity in multiple syngeneic mouse models [55] (Table 1). Intriguingly, Lai et al. reported that USP4, which is distinct from the abovementioned DUBs, exerts tumor-suppressive effects in the context of cancer progression [56]. TAM-secreted inflammatory cytokines (e.g., IL-1β and TNF-α) induce Snail expression in lung cancer cells, epigenetically downregulating USP4 to promote drug resistance, stemness and PD-L1 expression [56]. Hence, restoring USP4 levels is important for suppressing cancer progression. Hypomethylating agents or nanomedicine-based protein delivery agents are available therapeutic agents [57]; however, their application requires further investigation.

Although targeting oncogenic DUBs to suppress TAM-elicited tumor-promoting effects has potential, several urgent problems warrant consideration. First, strategies to specifically target TAMs with dysregulated DUBs but not macrophages resident in normal tissues or peripheral circulation should be noticed [40]. Second, conventional binary classification of macrophages is oversimplified for accurate assessment of phenotypes. According to single-cell sequencing, TAMs may coexpress M1- and M2-related genes and exhibit a spectrum of phenotypes [58]. Whether the protein level or activity of DUBs determines the degree of macrophage polarization or TAM heterogeneity within the TME remains obscure and requires further investigation.

### DUBs-induced MDSC immunosuppressive activity and recruitment

MDSCs constitute a heterogeneous population of activated immune cells with immunosuppressive abilities that are physiologically responsible for T-cell tolerance [59, 60]. However, infiltrating MDSCs are capable of triggering immune escape via several approaches. For instance, MDSCs can release immunosuppressive cytokines (e.g., IL-10 and TGF-β) or promote Treg infiltration, eventually suppressing CD8^+^ T-cell activity [61, 62]. In addition, MDSC recruitment decreases the efficacy of ICB, and the abundance of MDSCs relative to that of infiltrating CD8^+^ T cells determines the effectiveness of immunotherapies [36, 63]. These findings underscore the crucial role of MDSCs in immunosuppression. MDSCs are primarily divided into two subsets: granulocytic MDSCs (G-MDSCs) and monocytic MDSCs (M-MDSCs) [64]. A recent study suggested that USP12 enhances the infiltration and suppressive function of M-MDSCs concomitant with PD-L1 upregulation through the deubiquitination and stabilization of NF-κB p65 [26]. In particular, high expression of PD-L1 on MDSCs even causes apoptosis of T cells [65]. Hence, targeting USP12 might reduce the activity of MDSCs and further restore CD8^+^ T-cell cytotoxicity to synergize with ICB. Furthermore, Li et al. showed that PDAC-intrinsic USP22 changes the transcriptome to enable the recruitment of MDSCs [36]. A20, which belongs to the OTU subfamily, is a well-characterized negative regulator of NF-κB signaling and TNF-mediated apoptosis [66]. Intriguingly, previous findings suggested that A20 was overexpressed in MDSCs, while inhibition of A20 not only reduced the infiltration of MDSCs but also induced MDSC apoptosis via JNK activation, potentiating immunotherapy [67]. As mentioned earlier, both USP10 and OTUD5 are responsible for YAP stabilization and activation of downstream signaling pathways [23, 25]. High expression of YAP was shown to upregulate the expression of chemokines involved in MDSC recruitment, including CXCL5, CXCL1/2, and CCL2, in vitro and in vivo [43, 68]. However, whether blockade of USP10 or OTUD5 can reactivate CD8^+^ T-cell-mediated cytotoxicity against tumors in a MDSC-dependent manner remains unclear and requires further exploration.

### The role of DUBs in CD4^+^ T cell differentiation

**Fig. 2:**
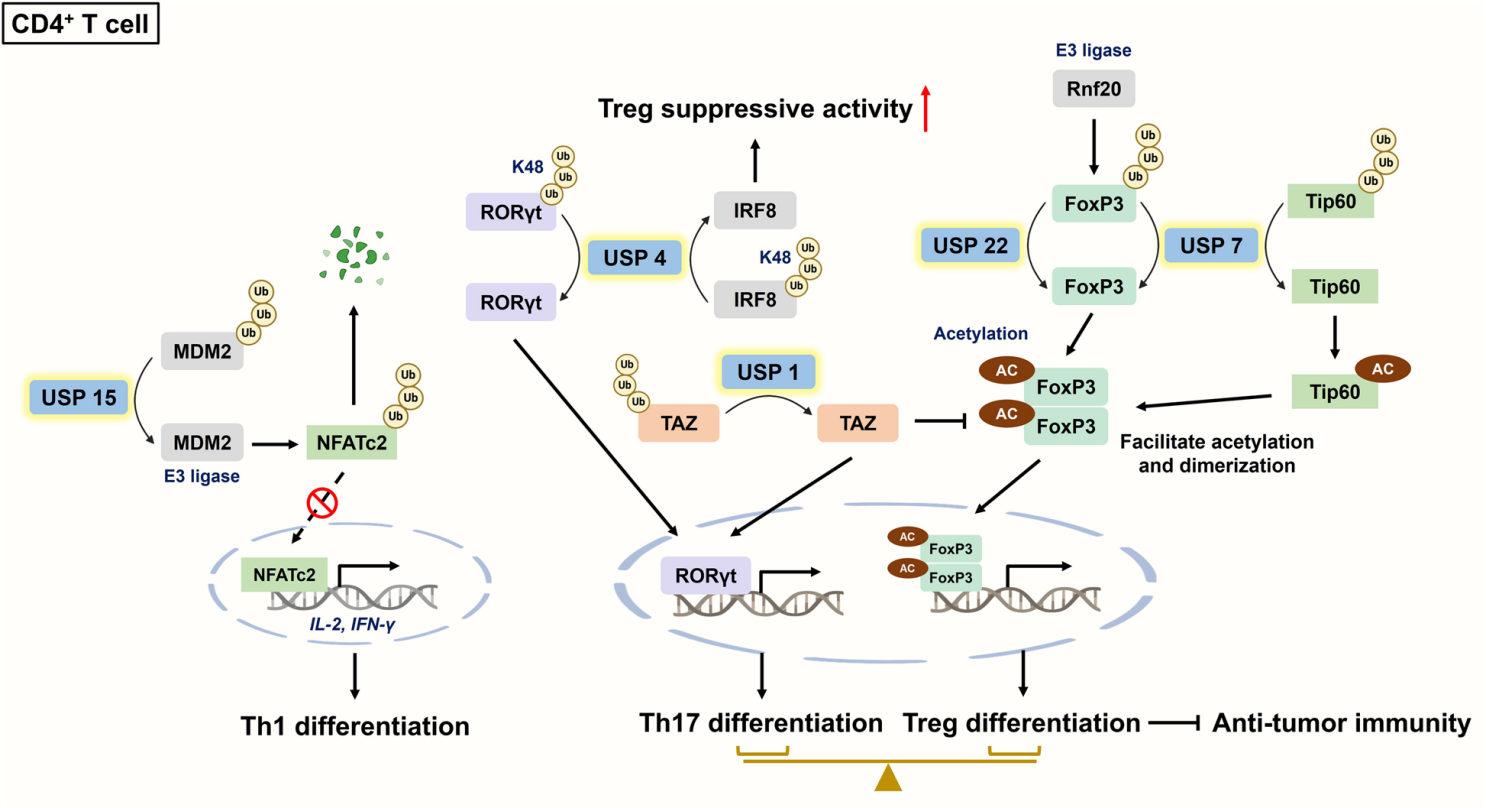
The roles of DUBs in modulating CD4^+^ T-cell differentiation. DUBs can regulate CD4^+^ T-cell differentiation into specific subsets. This not only highlights their roles in immunomodulation but also their implications in cancer immunotherapy. USP15 stabilizes MDM2 to enhance NFATc2 degradation, which downregulates IL-2 and IFN-γ and inhibits Th1 differentiation. Intriguingly, USP4 plays a dual role in CD4^+^ T-cell lineage commitment. On the one hand, it deubiquitinates RORγt to facilitate Th17 differentiation; on the other hand, it stabilizes IRF8 to augment the suppressive activities of Tregs, circumventing CD8^+^ T-cell function and infiltration. Both USP22 and USP7 induce Treg differentiation. USP22 counteracts E3 ligase Rnf20-mediated polyubiquitination of FoxP3; USP7 simultaneously deubiquitinate and stabilize FoxP3 and Tip60. These processes facilitate dimerization and acetylation of FoxP3, but are counteracted by USP1-mediated deubiquitination of TAZ. FoxP3: Forkhead box protein P3; IRF8: interferon regulatory factor 8; MDM2: murine double minute 2; NFATc2: nuclear factor of activated T cells c2; Rnf20: ring finger protein 20; RORγt: RAR-related orphan receptor-γ; Tip60: Tat-interactive protein 60

Activated CD4^+^ T lymphocytes undergo lineage commitment via the regulation of specific cytokines and corresponding transcription factors [69]. Differentiated CD4^+^ T cells primarily consist of several subsets, including T helper 1 (Th1), Th2, and Th17 cells and Tregs [70]. Recent studies have indicated that DUBs can regulate CD4^+^ T-cell activation and even determine their differentiation into specific subsets. Murine double minute 2 (MDM2), a well-known negative modulator of p53 [71], can be deubiquitinated by USP15 to degrade nuclear factor of activated T cells c2 (NFATc2), which is crucial for T-cell-related cytokine (e.g., IL-2 and IFN-γ) expression **(**Fig. 2**)**. Loss of USP15 promoted Th1 differentiation, but this phenomenon was not observed in CD8^+^ T cells, as explained by the relatively low expression of MDM2 in CD8^+^ T cells. Intriguingly, *Usp15*^−/−^ mice challenged with B16 cells surprisingly exhibited increased infiltration and activation of CD8^+^ T cells, which was probably facilitated by indirect effects of CD4^+^ T cells secreting IFN-γ [72]. In addition to USP15, USP7 was also found to deubiquitinate MDM2 [73]; furthermore, a previous study demonstrated the physical interaction between USP7 and USP15 [74]. Nevertheless, whether USP7 can function as a USP15 to modulate Th cell differentiation warrants further research.

Th17 cells serve as ‘Jekyll and Hyde’ cells in tumor progression due to their strong plasticity [75, 76]. Despite the controversial role of Th17 cells in tumor progression, Tregs are involved mostly in self-tolerance and secrete immunosuppressive cytokines, including IL-10 and TGF-β, to exert protumor effects. TGF-β secretion reportedly inhibits the infiltration of NK cells, macrophages and DCs; moreover, increased Treg infiltration into the TME indicates a poor prognosis [77, 78]. An imbalance of Th17 cells and Tregs is strongly associated with cancer progression [79, 80]; strikingly, USP1 is reported to be a vital regulator of the differentiation of Th17 cells and Tregs. CD4^+^ T cell-intrinsic USP1 facilitates Th17 differentiation rather than Treg differentiation through the activation of RAR-related orphan receptor-γ (RORγt) and proteasomal degradation of Forkhead box protein P3 (FoxP3) in a transcriptional coactivator with PDZ-binding motif (TAZ)-dependent manner [81]. RORγt and FoxP3 are specific transcription factors that regulate Th17 cells and Tregs, respectively. Consistently, a previous study reported that TAZ not only acts as a critical coactivator of RORγt but also promotes FoxP3 degradation by reducing its acetylation [82]. Furthermore, USP4 is capable of driving Th17 cell differentiation by catalyzing the K48-linked deubiquitination of RORγt [83] **(**Fig. 2**)**. Lineage commitment to a specific subset simultaneously inhibits the gene expression of the opposing CD4^+^ T-cell subset [69]. ML323, a selective USP1 inhibitor, was suggested to exert antineoplastic effects on several cancer types. For example, ML323 induces cell cycle arrest in esophageal squamous cell carcinoma [84] and sensitizes renal cell carcinoma (RCC) cells to apoptosis through death receptor 5 upregulation and survivin degradation [85]. Unfortunately, immunodeficient xenograft mouse models were used to investigate the role of USP1 in RCC progression [85]; hence, the effect of Th17/Treg imbalance on tumor progression was overlooked. Nevertheless, ML323 potently regulates the Th17/Treg imbalance, demonstrating its potential for cancer treatment.

Emerging evidence has revealed that Treg-intrinsic DUBs can regulate the suppressive function of Tregs, suggesting that they are significant therapeutic targets for augmenting antitumor immunity. Cortez and associates employed the CRISPR-based loss-of-function platform to screen candidate proteins that regulate FoxP3 expression in mouse Tregs. The results revealed that USP22 can promote FoxP3 deubiquitination and stabilization, which is counteracted by the E3 ligase ring finger protein 20 (Rnf20). As expected, USP22 deficiency dramatically impaired Treg suppressive function but increased the proportion of IFN-γ^+^GrB^+^ CD8^+^ T cells and significantly inhibited tumor growth in multiple cancer models [86]. Additionally, USP4 can facilitate K48-limked deubiquitination of interferon regulatory factor 8 (IRF8) to enhance the suppressive activity of Tregs [87]. IRF8 is a transcription factor, and its deficiency impairs the Treg-elicited inhibitory effect on the Th1-mediated immune response [88]. Acetylation and dimerization of FoxP3 are important for Treg function [89, 90]. USP7 was shown to indirectly facilitate FoxP3 acetylation and dimerization by stabilizing Tat-interactive protein 60 (Tip60, a histone acetyltransferase that mediates FoxP3 acetylation) **(**Fig. 2**)**. Pharmacological inhibition or genetic deletion of USP7 particularly compromises the function of Tregs but not that of other effector T cells, suggesting the selectivity of this target [91]. Although anti-CTLA4 mAbs potently trigger antibody-dependent cytolysis of Tregs, there are no specific antibodies that target Tregs [92]. USP7 inhibitors (e.g., 564) not only have dominant effects on Tregs but also reportedly increase the efficacy of ICB and antitumor vaccines in vivo [91] **(**Table 1**)**. Overall, these findings indicate that blockade of USP7 may be a potential strategy for selectively targeting Tregs to avoid unwanted effects on other infiltrating effector T cells.

### The inhibitory effects of DUBs on CD8^+^ T cell infiltration and function

**Fig. 3:**
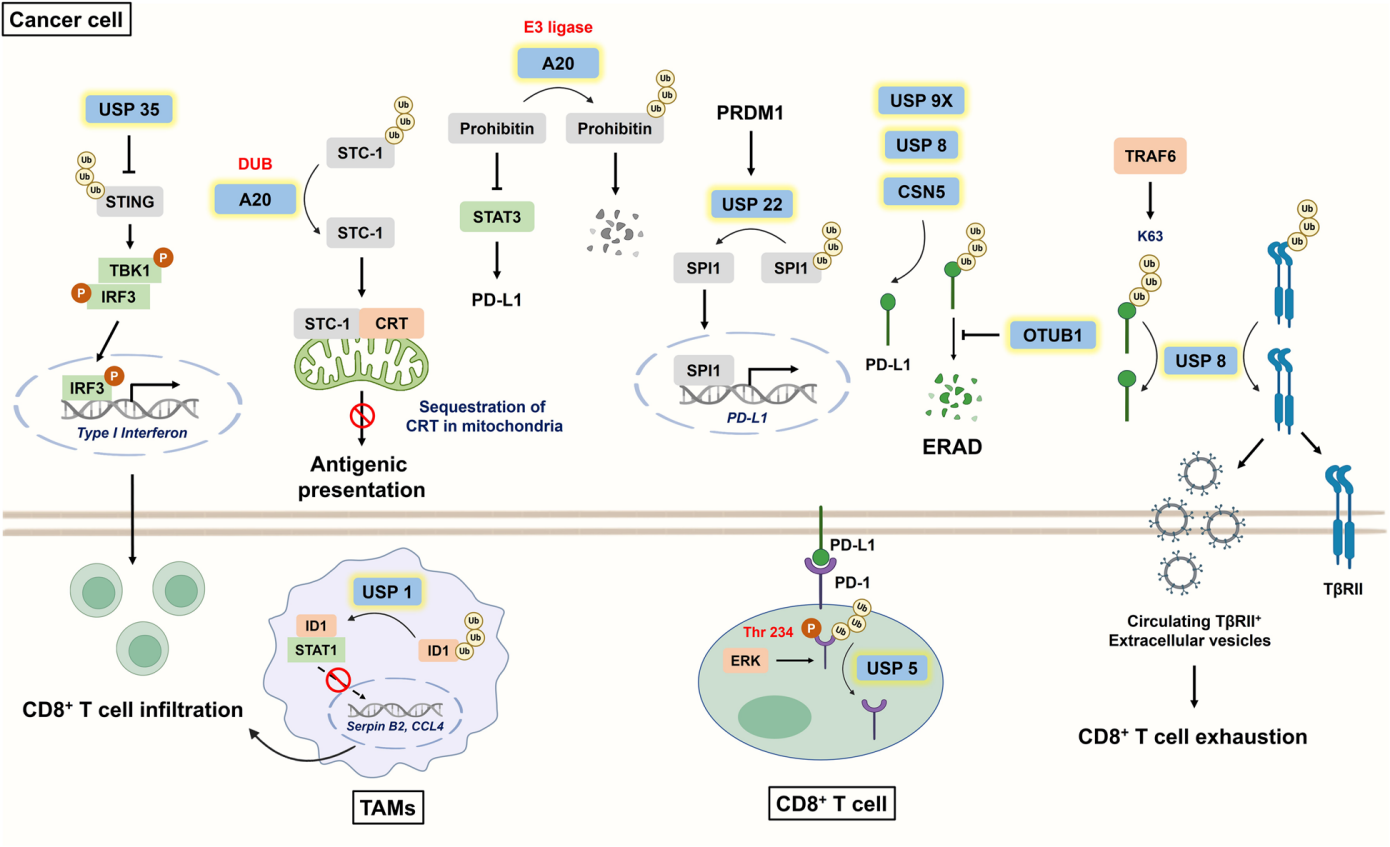
The involvement of DUBs in suppressing CD8^+^ T-cell infiltration and function. USP35 inhibits STING polyubiquitination and activation, ultimately downregulating IRF3-mediated type I interferon, which is important for CD8^+^ T-cell recruitment. Additionally, TAM-intrinsic USP1 deubiquitinates ID1 to prevent the nuclear translocation of STAT1 and the expression of CD8^+^ T-cell-recruiting factors (e.g., Serpin B2 and CCL4). A20 deubiquitinates and upregulates STC-1 to restrain the translocation of CRT from mitochondria to the plasma membrane, subsequently impeding antigenic presentation. With respect to CD8^+^ T-cell exhaustion, the PD-L1/PD-1 interaction has been extensively studied. Intriguingly, A20 upregulate PD-L1 via E3 ligase rather than deubiquitinating activity. Mechanistically, it ubiquitinates prohibitin but in turn induces STAT3/PD-L1 signaling

CD8^+^ T cells are the main effector cells involved in the antitumor response. Mechanistically, CD8^+^ T cells recognize antigens presented by tumor cells and subsequently trigger apoptosis to eliminate target cells via the secretion of perforin/granzyme or the initiation of Fas-mediated death signals [93, 94]. However, low infiltration and exhaustion of CD8^+^ T cells markedly reduce the durability of the ICB response [95]. The response to PD-L1/PD-1 blockade is determined by several factors, including the degree of antigen presentation, the expression levels of immune checkpoint proteins, the infiltration of cytotoxic T lymphocytes (CTLs), and the activation of CTLs (e.g., IFN-γ signaling) [96, 97]. A recent study revealed that DUBs can impair antigenic presentation and further T cell activation, which to some degree compromises the efficacy of ICB therapy. Calreticulin (CRT), an eat-me signal displayed on the plasma membrane, is recognized by antigen-presenting cells (APCs), and then tumor-derived antigens are presented to prime and activate specific T cells [98]. A20 upregulated stanniocalcin-1 (STC-1) to restrain the translocation of CRT from mitochondria to the plasma membrane, subsequently impeding antigenic presentation (Fig. 3); however, A20 inhibition potentially enhanced the efficacy of anti-PD-1 blockade in CRC [99].

With respect to CTL recruitment, USP35 silencing counteracts stimulator of interferon genes (STING) deubiquitination, which facilitates STING-mediated upregulation of type I interferons and CD8^+^ T-cell infiltration in ovarian cancer [100]. Notably, STING polyubiquitination is crucial for its activation, further recruitment of TANK-binding kinase 1 (TBK1) and phosphorylation of interferon regulatory factor 3 (IRF3) [101, 102] **(**Fig. 3**)**. Notably, USP35 knockdown sensitized tumors to cisplatin, and cisplatin treatment was reported to enhance CTL infiltration in vivo via cGAS/STING signaling [100, 103]. As mentioned previously, USP1 inhibits CTL infiltration by deubiquitinating ID1 and suppressing the secretion of recruitment-related factors [19]. OTU deubiquitinase ubiquitin aldehyde binding 1 (OTUB1) was reported to govern immunocyte infiltration and is recognized as a poor prognostic indicator for multiple tumors of the digestive system [104]. This is probably because OTUB1 can augment TGFβ signaling by preventing the ubiquitination of mothers against decapentaplegic homolog 2 and 3 (SMAD2/3) [105], which potently contributes to immune exclusion from tumors [18].

The exhaustion of effector T cells is primarily mediated by interactions between inhibitory checkpoint molecules and their corresponding inhibitory receptors. In particular, the PD-L1/PD-1 axis has been extensively studied. Cumulative studies have revealed that DUBs can facilitate the impairment of CD8^+^ T-cell function mainly by directly deubiquitinating or indirectly transcriptionally upregulating immune checkpoints (illustrated in Fig. 3). Ubiquitinspecific peptidase 9, X-linked (USP9X) was shown to directly deubiquitinate PD-L1 in oral squamous cell carcinoma [106]; moreover, CSN5 facilitates PD-L1 deubiquitination to enhance its stability in several malignancies, such as BC and GC [33, 107, 108], suggesting that targeting CSN5 might be globally effective across multiple tumors. In addition to curcumin, berberine (BBR), a natural product and traditional Chinese medicine, can downregulate PD-L1 in non-small cell lung cancer (NSCLC) to strengthen the tumor-infiltrating T-cell-mediated immune response by reducing CSN5 activity [107, 109] **(**Table 1**)**. Although low-dose BBR (< 20 µM) cannot directly eliminate NSCLC cells, it potently promotes the immune clearance of tumors [107]. Shikonin, a naphthoquinone compound, can also induce PD-L1 degradation by inhibiting NF-κB/CSN5 expression in PC [110]. Zhu and associates suggested that OTUB1 can remove K48-linked ubiquitin chains of PD-L1 to prevent newly synthesized PD-L1 from undergoing endoplasmic reticulum-associated protein degradation [24]. OTUB1 ablation not only enhances CTL recruitment but also increases IFN-γ levels to profoundly enhance antitumor immunity in vivo [24, 111]. Within CD8^+^ T cells, extracellular signal-regulated kinase (ERK) facilitates PD-1 phosphorylation at Thr234, which enhances its interaction with USP5 to deubiquitinate and increase PD-L1 levels, while USP5 inhibition results in elevated levels of IFN-γ and GrB. Moreover, combined treatment with EOAI34 (a USP5 inhibitor) and trametinib (a MEK inhibitor) in CT26 tumor-bearing mice slowed tumor growth and increased CD8^+^ T-cell infiltration [20] **(**Table 1**)**.

For the transcriptional upregulation of PD-L1, Mao et al. suggested that ubiquitin C-terminal hydrolase L1 (UCHL1) promoted PD-L1 levels by activating the Akt/NF-κB p65 axis [112]. Strikingly, A20 was reported to upregulate PD-L1 via E3 ligase rather than deubiquitinating activity in melanoma. Indeed, A20 possesses both E3 ligase and DUB functions and is referred to as a ubiquitin-editing enzyme. Mechanistically, A20 facilitates the ubiquitination of prohibitin but in turn induces signal transducer and activator of transcription 1 (STAT3)/PD-L1 signaling (Fig. 3); however, A20 inhibition invigorates exhausted CD8^+^ T cells [113]. Positive regulatory domain containing 1 (PRDM1) functions as a double-edged sword in hepatocellular carcinoma (HCC) progression. *PRDM1* overexpression can decrease the proliferative capacity of HCC cells but transcriptionally upregulate PD-L1 via the USP22/SPI1 axis, subsequently facilitating T-cell exhaustion. Mechanistically, SPI1 serves as a transcription factor for *PD-L1* and is deubiquitinated by USP22 (Fig. 3). Indeed, *PRDM1* overexpression failed to retard tumor growth in immunocompetent mice. Based on these findings, delivery of the *PRDM* gene via therapeutic vectors (e.g., adeno-associated viruses) has been proposed to synergize with anti-PD-1 or PD-L1 mAbs; however, its safety and efficacy require extensive investigation [114].

Long non-coding RNAs (lncRNAs) and circular RNAs (circRNAs), previously recognized as junk RNAs, can serve as competitive endogenous RNAs (ceRNAs) to sponge miRNAs and indirectly regulate downstream gene expression [115]. Recent studies have shown that both lncRNAs and circRNAs can increase PD-L1 levels by sequestering specific miRNAs that negatively target DUBs. For instance, lncRNA GATA3-AS1 can sequester miR-676-3p to promote the CSN5 level, which deubiquitinates PD-L1 and facilitates immunosuppression in TNBC [108]. Similarly, the lncRNA KCNQ1OT1 facilitates CD8^+^ T-cell exhaustion through the miR-30a-5p/USP22/PD-L1 axis. The lncRNA KCNQ1OT1 is highly expressed in tumor-derived exosomes, which are believed to promote PD-L1 levels and immune evasion of neighboring CRC cells [116]. Liu et al. demonstrated that circIGF2BP3 upregulated PD-L1 in an OTUB1-dependent manner by sponging miR-328-3p and miR-3173-5p; accordingly, circIGF2BP3 inhibition sensitized NSCLC to anti-PD-1 blockade concomitant with increased activation of CTLs in vivo [117].

Strikingly, DUB was reported to inhibit immunosurveillance in BC via an extracellular vesicle (EV)-dependent manner [118]. Mechanistically, USP8 deubiquitinated and increased the level of TGF-β type II receptor (TβRII) in both the plasma membrane and in EVs, and circulating TβRII^+^ EVs mediated CD8^+^ T-cell exhaustion **(**Fig. 3**)**. After USP8 depletion, tumor-derived TβRII^+^ EVs reached nearly undetectable levels; patients with high USP8 expression were resistant to neoadjuvant chemotherapy, suggesting its potential as a biomarker. Although previous studies revealed that USP4 and USP8 can stabilize TGF-β type I receptor (TβRI) [119, 120], targeting TβRII might be promising because it is relatively unstable, and its stability determines the duration of TGF-β signaling. Notably, selective inhibition of TβRII by targeting USP8 should be carefully considered because knockout of TβRII in BC results in enhanced metastasis and MDSC-elicited suppressive effects [121].

Notably, two recent studies illustrated the paradoxical role of USP8 in modulating PD-L1 levels **(**Fig. 3**)**. On the one hand, USP8 deubiquitinates PD-L1 in PDAC, while combined treatment with a USP8 inhibitor and an anti-PD-L1 antibody enhances the infiltration of IFN-γ^+^ TNF-α^+^ CD8^+^ T cells and effectively suppresses liver metastasis by increasing antitumor immunogenicity [21]. On the other hand, USP8 instead downregulates PD-L1 in lung cancer and CRC, and USP8 blockade increases the efficacy of ICB [22]. Mechanistically, the E3 ligase tumor necrosis factor receptor-associated factor 6 (TRAF6) facilitates K63-linked polyubiquitination of PD-L1, whereas this effect is counteracted by USP8 since USP8 preferentially cleaves K63-linked ubiquitin rather than K48-linked ubiquitin [122]. Generally, K48-linked ubiquitination initiates proteasomal degradation, but K63-linked ubiquitination is recognized as a nondegradative process [72]. USP8 inhibition by DUB-IN-2 not only upregulated MHC I via NF-κB signaling but also increased PD-L1 levels by maintaining K63-linked polyubiquitination to improve the sensitivity of cells to ICB in vivo [22] **(**Table 1**)**. The contrasting outcomes could be explained by differences in cancer types and underlying mechanisms. Moreover, the antagonistic effect of USP8 against TRAF6-mediated K63-linked polyubiquitination was not investigated in PDAC [21, 22]. Although targeting USP8 has the potential to synergize with cancer immunotherapy despite the completely different results obtained for PD-L1 expression, the treatment window for combination therapy should be carefully evaluated because the half-life of DUB-targeted substrates might significantly determine the efficacy of ICB.

It is worth examining the relationship between PD-L1 expression and ICB efficacy. Altered expression of PD-L1 (either downregulation or upregulation) has been shown to improve the efficacy of immunotherapy. The former releases the brake on immunosurveillance; however, the latter turns immune “cold” tumors into immune “hot” tumors to increase ICB sensitivity [123]. Thus, PD-L1 expression alone is not a reliable biomarker for predicting the response to an anti-PD-L1/PD-1 mAb. Indeed, some patients with PD-L1 positivity exhibit no response to therapy, but some patients with PD-L1 negativity may respond [124, 125]. Hence, the significance of T-cell infiltration should be considered when predicting the tumor response to ICB. Specifically, inducible PD-L1 expression by infiltrating T cells indicates the presence of activated T cells because IFN-γ reportedly promotes PD-L1 transcription. PD-L1 expression in this setting likely mediates a strong response to ICB and reinvigorates T cells. Additionally, PD-L1-negative tumors without T-cell infiltration could also respond to ICB if PD-L1 expression is induced by a combination treatment that enhances T-cell infiltration [125, 126].

USP22 can deubiquitinate SPI1 to transcriptionally upregulate PD-L1. Moreover, USP9X, USP8, and CSN5 can deubiquitinate PD-L1, and OTUB1 increase PD-L1 levels by preventing new synthetic PD-L1 from entering the ERAD. Strikingly, USP8 plays a dual role in controlling PD-L1 levels in distinct cancer types. In PDAC, USP8 directly removes K48-linked ubiquitin-bound PD-L1 to increase its stability. Conversely, USP8 preferentially cleaves TRAF6-mediated K63-linked ubiquitination, which instead downregulates PD-L1 in CRC. Within CD8^+^ T cells, PD-1 phosphorylation at Thr234 via ERK signaling facilitates its interaction with USP5 to promote protein stability and further promote T-cell exhaustion. CRT: Calreticulin; CSN5: COP9 signalosome 5; ERAD: endoplasmic reticulum-associated protein degradation; ID1: inhibitor of differentiation 1; IRF3: interferon regulatory factor 3; OTUB1: OTU deubiquitinase, ubiquitin aldehyde binding 1; STAT1: signal transducer and activator of transcription 1; STAT3: signal transducer and activator of transcription 3; STC-1: stanniocalcin-1; STING: stimulator of interferon genes; TBK1: TANK-binding kinase 1; TRAF6: tumor necrosis factor receptor-associated factor 6.

### CAFs-mediated suppression of CD8^+^ T cell function in a DUB-dependent manner

CAFs are located in the stroma and are involved in several cancer-related processes, including invasion, angiogenesis, metastasis, chemoresistance and immunosuppression [127–129]. CAFs facilitate immune escape not only by forming a physical barrier but also by releasing various cytokines and chemokines to inhibit the recruitment and activity of effector immune cells [28]. For instance, CAF-derived C-X-C motif chemokine ligand 12 (CXCL12) sequesters CD8^+^ T cells, which reduces CD8^+^ T-cell infiltration in the juxtatumoral compartment (identified as < 100 μm from the tumor) [17, 130]. A recent study suggested that CAF-derived CXCL12 promoted PD-L1 expression in bladder cancer. Mechanistically, the CXCL12/CXCR4 axis upregulates DUB cylindromatosis (CYLD) via JAK2/STAT3 signaling to facilitate p62 deubiquitination and accumulation, which suppresses the autophagic degradation of PD-L1 [131]. Consistent with the findings of previous studies, the inhibition of autophagic flux in turn promoted PD-L1 expression in GC [132]. However, CYLD was also reported to serve as a tumor-suppressive DUB by negatively modulating the oncogenic activation of NF-κB [133], revealing its dual role in tumor progression.

**Table 1:** Potential small molecules that target DUBs to relieve immunosuppression and suppress tumorigenesis

DUB subfamily	DUB	Inhibitor/Compound	Cell-intrinsic DUB	Mechanism / Effect / Implication	Reference(s)
JAMMs	CSN5	Berberine	NSCLC	1. Selectively target Glu76 residue of CSN5 to decrease PD-L1 levels 2. Suppress MDSCs and Tregs activation but enhance effector T cell function and infiltration	[107]
		Curcumin	BC, NSCLC, Melanoma, CRC	1. Inhibit CSN5 activity to downregulate PD-L1 2. Synergize with anti-CTLA-4 mAb and promote effector T cell function	[55]
USPs	USP1	ML323	Macrophage	1. Inhibit USP1/ID1 signaling to upregulate CD8^+^ T-cell-recruiting factors (e.g., CCL4 and SerpinB2) 2. Increase CD8^+^ T cell infiltration and function and suppress colorectal liver metastasis 3. Improve CRC sensitivity to 5-FU and anti-CTLA-4 mAb	[19]
	USP5	EOAI34	CD8^+^ T-cell	1. Block USP5-mediated deubiquitination of PD-1 2. Combine with Trametinib (MEK inhibitor) to increase CD8^+^ T cell infiltration and exhibit growth inhibition in lung and colon cancer	[20]
	USP7	564	Treg	1. Reduce the recruitment and suppressive function of FoxP3^+^ Tregs 2. Improve the efficacy of antitumor vaccine and ICB in murine models bearing lung cancer or mesothelioma	[91]
		P5091	Macrophage	1. Activate p38 MAPK signaling to reprogram macrophage polarization towards M1 phenotype 2. Delay growth of lung cancer concomitant with increased M1 and CD8^+^ T cell infiltration	[52]
	USP8	9-Ethyloxyimino‐9 H‐indeno[1,2‐b]pyrazine‐2,3‐dicarbonitrile	BC	1. Decrease the stability of TβRII and circulating TβRII^+^ EVs to prevent CD8^+^ T cell exhaustion 2. Improve the efficacy of ICB but suppress metastasis	[118]
		DUBs-IN-2	Colon cancer and NSCLC	1. Increase PD-L1 levels through restoring TRAF6-mediated K63-linked ubiquitination 2. Promote MHC I expression and CD8^+^ T cell infiltration 3. Augment the tumor sensitivity to anti-PD-L1/PD-1 mAb	[22]
			PDAC	1. Decrease PD-L1 levels through directly targeting USP8-mediated deubiquitination of PD-L1 2. Synergize with anti-PD-L1 mAb to increase CD8^+^ T cell infiltration and function but suppress liver and lung metastasis	[21]
	USP14	IU1	Macrophage	1. Block USP14/SIRT-1/PGC-1α axis to metabolically reprogram TAMs 2. Inhibit EMT and tumor growth of GC	[45]

*BC* breast cancer, *CCL4* C-C motif chemokine ligand 4, *CRC,* colorectal cancer, *CSN5* COP9 signalosome 5, *EMT* epithelial–mesenchymal transition, *EVs* extracellular vesicles, *GC* gastric cancer, *ICB*, immune checkpoint blockade, *JAMMs* JAMM/MPN domain-associated metallopeptidases, *MDSCs,* myeloid-derived suppressor cells; *NSCLC* non-small cell lung cancer, *PGC-1α* peroxisome proliferator-activated receptor-γ coactivator-1α, *SIRT-1* sirtuin-1, *Tregs* regulatory T cells, *TβRII* TGF-β receptor II, *USPs* ubiquitin-specific proteases

## Discussion

The emergence of ICB has revolutionized cancer therapeutics and brought benefits for patients with hematological malignancies in recent years [11]. Unfortunately, the response rate to ICB is approximately 20% in patients with solid tumors [134], which is to some extent influenced by the heterogeneity of the TME [135]. Ubiquitination and deubiquitination are tightly regulated posttranslational modifications that maintain cellular homeostasis. Accumulating evidence has demonstrated the dysregulation of DUBs in cancer development and their involvement in modulating the TME for immunosuppression [21, 26, 36, 48]. Hence, a better understanding of the role of DUBs in the interplay between tumors and TME components is promising for cancer immunotherapy. In the following context, we elucidate the prospects and challenges of targeting DUBs in cancer immunotherapy.

With respect to prospects, first, the enzymes involved in the ubiquitination cascade have clinical implications in cancer [136]. However, DUBs seem to be better therapeutic targets than E3 ligases because the former possess conserved catalytic cysteines, while the latter lack identified catalytic residues [137]. Second, targeting DUBs might be feasible and have the potential to eliminate undruggable oncoproteins since their cellular homeostasis is strictly regulated by the ubiquitin‒proteasome system. For instance, YAP1 was reported to upregulate PD-L1 and even promote M2 polarization to drive immune evasion [23]. Unfortunately, targeting YAP1 is technically challenging due to the lack of known catalytic activity [138]. USP10 can deubiquitinate and stabilize YAP1, and its inhibitor Wu-5 was found to potently suppress leukemia [139, 140], exhibiting its potential for eliminating undruggable oncoproteins to restore immunosurveillance. Third, DUB inhibitors may be used as adjuvants with clinically prescribed treatments (e.g., chemotherapy and ICB) [36]. This not only reduces the dosage requirement of single agents but also decreases the probability of acquired resistance. Indeed, blockade of USP8 by DUBs-IN-2 synergizes with ICB to retard tumor growth [22]. As demonstrated previously, curcumin and BBR are suggested to function as ICB agents by inhibiting the CSN5/PD-L1 axis [55, 107]. Notably, compared to therapeutic antibodies, small-molecule inhibitors that target DUBs to regulate immune checkpoints might possess several advantages, including greater tissue penetration, a safer profile after oral administration, and favorable pharmacokinetics [141]. Notably, several DUB inhibitors are now potent and selective (e.g., FT671, a USP7 inhibitor with nanomolar potency) [142], and some of them are even being tested in clinical trials, which highlights their potential to synergize with ICB to augment antitumor immunity. For example, the Mission Therapeutics-developing USP30 inhibitor MTX652 has demonstrated a favorable pharmacokinetic profile during the phase I clinical trial. Furthermore, KSQ-4279, a USP1 inhibitor, in combination with a PARP inhibitor (e.g., olaparib) is in a phase I clinical trial to evaluate its safety and clinical activity in patients with advanced solid tumors (NCT05240898). Whether KSQ-4279 can act as an ML323 to synergize with an anti-CTLA-4 mAb to suppress CRC progression by inhibiting the USP1/ID1 axis requires investigation.

Although the abovementioned advantages highlight that DUB inhibitors are potential cancer therapeutics, several challenges and unsolved problems warrant attention and intensive research.

(i) Deubiquitination is an intracellular biological process; thus, unlike small-molecule compounds, therapeutic antibodies cannot bind DUBs [143]. Notably, a recent study demonstrated that dimeric IgA can effectively target intracellular oncoproteins to suppress tumor growth through the polymeric immunoglobulin receptor, which might be a solution for this obstacle [144].

(ii) Numerous DUBs can regulate identical targets (e.g., PD-L1); vice versa, the substrates of a specific DUB are not fully known. Hence, most first-generation DUB inhibitors are multitargeted, which likely affects nontarget pathways and causes toxicity. The USP14/ubiquitin C-terminal hydrolase L5 (UCHL5) inhibitor VLX1570 was reported to inhibit multiple myeloma (MM) progression in vivo [145]; unfortunately, a phase I/II clinical trial (NCT02372240) evaluating the safety and efficacy of VLX1570 and dexamethasone in patients with refractory MM was terminated due to intolerable toxicity [146]. Thus, the toxicity and pharmacokinetics of DUB inhibitors in vivo should be carefully considered despite the differential expression of DUB in tumors.
(iii) DUBs seemingly regulate distant molecular mechanisms in different malignancies or cell types. One salient example is USP8, which upregulates PD-L1 in PDAC via direct deubiquitination [21] but downregulates PD-L1 in CRC through counteracting TRAF6-mediated K63-linked polyubiquitination [22]. USP4 can serve as a double-edged sword in tumor progression because it functions as a tumor suppressor in lung cancer by indirectly circumventing stemness [56], while USP4 promotes TNBC metastasis by enhancing TGF-β signaling and inducing the suppressive activity of Tregs [87, 119]. Notably, most studies have focused on the oncogenic roles of DUBs rather than their tumor-suppressive activities. In addition, the therapeutic dose of and cellular sensitivity to DUB inhibitors are also major concerns. USP7 inhibition by P5091 can drive macrophage reprogramming, while reduced Treg suppressive function was not observed under USP7 blockade [52, 91]. It is speculated that the difference in sensitivity to a given DUB inhibitor between macrophages and Tregs might be a contributing factor.
(iv) Most DUBs possess similar catalytic pockets, and the conformations of their active sites change dynamically upon interaction with the ubiquitin moiety. Furthermore, the mechanisms regulating DUB enzymatic activity are complicated and involve substrate-mediated catalysis and allosteric regulation [147]. Notably, some DUBs preferentially target protein substrates harboring specific ubiquitin modifications, and diverse ubiquitin linkages (e.g., branched or mixed heterotypic ubiquitin chains and ubiquitin-like modifiers) might confer complexity to these client proteins [148]. For these reasons, highly selective targeting of DUBs is intractable. Nevertheless, Turnbull and colleagues identified FT671, a noncovalent USP7 inhibitor with nanomolar potency, which exhibits specificity and high affinity because it targets the dynamic pocket adjacent to the catalytic center and is distinguished from other DUB inhibitors [142].
(v) Activity-based probes (ABP) and fluorogenic substrates are commonly used tools for detecting DUB activity and screening DUB inhibitors. For example, Ub-AMC (7-amido-4-methylcoumarin) is used to measure the hydrolysis of ubiquitin linkages in the presence of DUB inhibitors [149]; however, fluorescence interference by small molecules has been reported. DUBs remove ubiquitin moieties via reactive thiol groups, which might lead to false-positive results since most inhibitor screening assays involve alkylating or nonspecific redox agents [74]. Moreover, high-throughput biochemical assays are generally limited by the low-hit rate and mere assessment of catalytic sites. The introduction and drawbacks of emerging screening technologies were elaborately discussed in a previous review [143]. Recently, Chan and associates took advantage of a structure-guided approach to accelerate the development of DUB inhibitors. Paired with activity-based protein profiling, the authors tailored the DUB-focused covalent library chemically diversified to target multiple discrete regions near the catalytic site and eventually identified selective inhibitors against the endogenous DUBs and a probe for the less-investigated DUB VCPIP1 with nanomolar potency [150]. Overall, obtaining an in-depth understanding of the biology of DUBs and optimizing screening technologies are important for the discovery of effective DUB inhibitors.

## Conclusion

Deubiquitination is an important posttranslational modification that strictly regulates cellular protein turnover. Emerging studies have indicated that dysregulation of DUBs can not only promote tumorigenesis but also participate in cellular interplay within the TME to facilitate immune escape, which makes DUBs promising therapeutic targets. In this review, we first comprehensively illustrated the roles of DUBs in the dynamic crosstalk between tumors, immune cells, and stromal cells. In addition, we discussed numerous DUB inhibitors that potently reverse immunosuppression. Finally, both the advantages and urgent problems associated with targeting DUBs for cancer treatment are discussed. In conclusion, an in-depth understanding of the biological characteristics of DUBs and the exploration of efficient DUB inhibitors by optimizing the present screening assays will be conducive for the development of DUB-targeting drugs.

## Data Availability

No datasets were generated or analysed during the current study.

## Abbreviations

BBR: berberine
BC: breast cancer
CAFs: cancer-associated fibroblasts
circRNAs: circular RNAs
CRC: colorectal cancer
ceRNAs: competitive endogenous RNAs
CSN5: COP9 signalosome 5
CYLD: cylindromatosis
CTLs: cytotoxic T lymphocytes
DUBs: deubiquitinating enzymes
FoxP3: Forkhead box protein P3
Gal-9: galectin-9
GC: gastric cancer
GrB: granzyme B
ICB: immune checkpoint blockade
ID1: inhibitor of differentiation 1
IRF3: interferon regulatory factor 3
IRF8: interferon regulatory factor 8
JAMMs: JAMM/MPN domain-associated metallopeptidases
LLC: Lewis lung cancer
lncRNAs: long noncoding RNAs
MJDs: Machado–Joseph disease protein domain proteases
MINDYs: motif interacting with Ub-containing novel DUBs
MDSCs: myeloid-derived suppressor cells
NK: natural killer
NSCLC: non-small cell lung cancer
NFATc2: nuclear factor of activated T cells c2
OTUD5: OTU deubiquitinase 5
OTUB1: OTU deubiquitinase, ubiquitin aldehyde binding 1
OTUs: ovarian tumor proteases
PDAC: pancreatic ductal adenocarcinoma
RORγt: RAR-related orphan receptor-γ
Tregs: regulatory T cells
STING: stimulator of interferon genes
TBK1: TANK-binding kinase 1
Tip60: Tat-interactive protein 60
TβRII: TGF-β type II receptor
TNBC: triple-negative breast cancer
TME: tumor microenvironment
TAMs: tumor-associated macrophages
TRAF6: tumor-necrosis factor receptor-associated factor 6
UCHs: ubiquitin carboxy-terminal hydrolases
USPs: ubiquitin-specific proteases
YAP1: yes-associated protein 1
ZUFSPs: Zn-finger and UFSP domain proteins
